# Supplementary material for: Insights into the Origin of Activity Enhancement via Tuning Electronic Structure of Cu2O towards Electrocatalytic Ammonia Synthesis
Source: Molecules. 2024 May 11;29(10):2261. doi: 10.3390/molecules29102261 (PMC11124335; doi:10.3390/molecules29102261)
Supplement: Supplementary file 1 [file molecules-29-02261-s001.zip › molecules-2984246-supplementary.pdf]

Supporting Information

Insights into the Origin of Activity Enhancement via Tuning Electronic Structure of Cu_2O towards Electrocatalytic Ammonia Synthesis

Meimei Kou, Ying Yuan, Ruili Zhao, Youkui Wang, Jiamin Zhao *, Qing Yuan * and Jinsheng Zhao *

School of Chemistry and Chemical Engineering, Liaocheng University, Liaocheng 252059, China; kmm2018705344@163.com (M.K.); yuanyingsdlc@163.com (Y.Y.); zhao1784308@163.com (R.Z.); m19861904240@163.com (Y.W.)

* Correspondence: zhaojiamin08031223@163.com (J.Z.); yuanqing@lcu.edu.cn (Q.Y.); j.s.zhao@163.com (J.Z.)

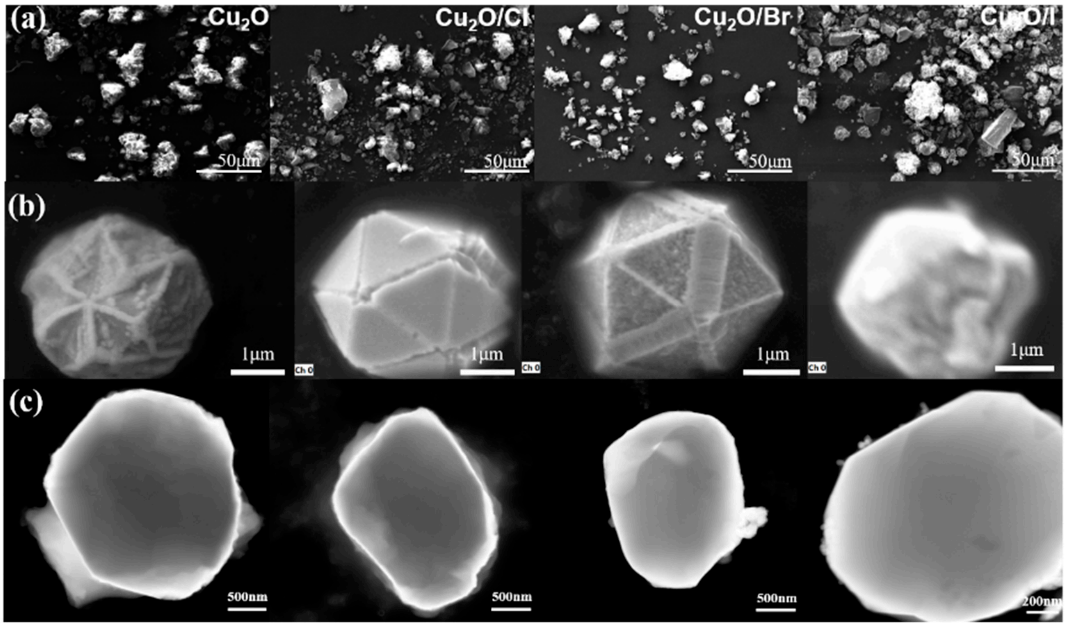

Figure S1. (a) and (b) represent SEM images of Cu_2O and Cu_2O/X (X = Cl, Br, I) at 50 μm and 1 μm; (c) TEM images of Cu_2O and Cu_2O/X (X = Cl, Br, I).

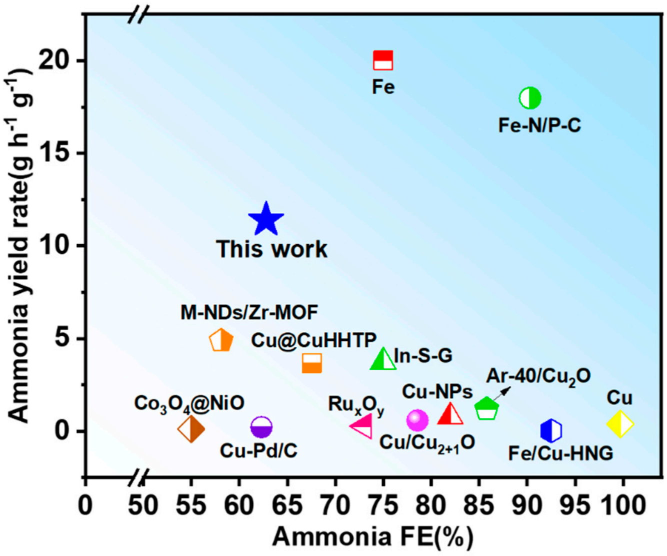

Figure S2. Comparison of electrocatalytic NO_3^-RR performance for Cu_2O/Br with other electrocatalysts under ambient conditions.

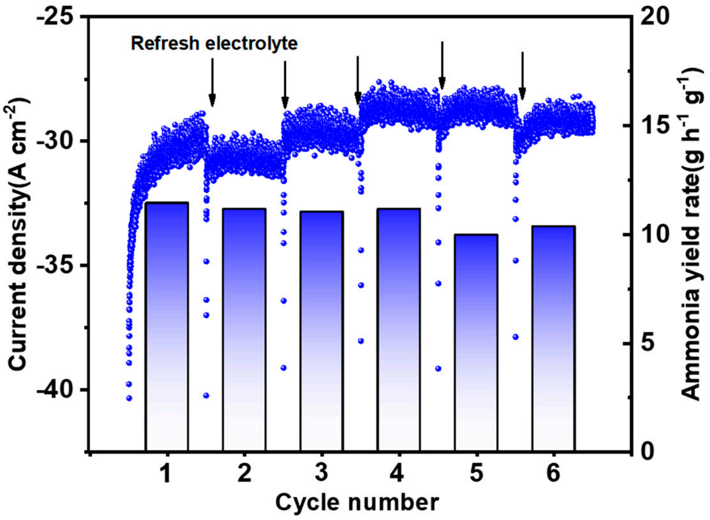

Figure S3. Stability test of ammonia electrosynthesis on Cu_2O/Br at -1.0 V over six continuous cycles (30min/cycle).

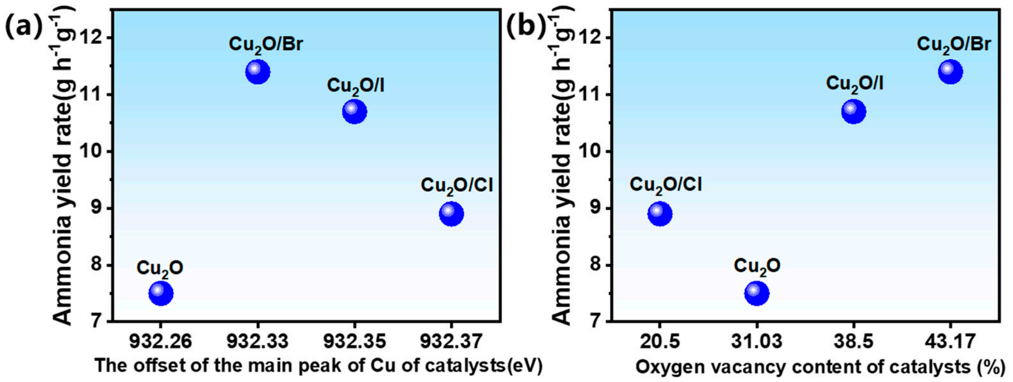

Figure S4. Plot of catalysts Cu main peak offset and oxygen vacancy content versus ammonia yield rate.

Table S1. The concentration of each halogen in the Cu_2O-halogen catalysts.

Catalyst	halogen	Atomic (%)
Cu_2O/Br	Br	3.27
Cu_2O/Cl	Cl	1.83
Cu_2O/I	I	3.44

Table S2. Comparison of electrocatalytic NO_3^-RR performance for Cu_2O/Br with other electrocatalysts under ambient conditions.

**Catalyst**	**Electrolyte**	**Faradaic efficiency (%)**	**NH_3 Yield rate ($g\ h^{-1}\ g^{-1}$)**	**Ref.**
**Cu_2O/Br**	**0.1M $KHCO_3$+ 0.1M KNO_3**	**62.78**	**11.4**	**This work**
Fe-N/P-C	0.1M KOH+ 0.1M KNO_3	90.3	17.98	1
Fe single atom	0.1M K_2SO_4+ 0.5M KNO_3	75	20	2
Cu-Pd/C	0.1M KOH+ 0.01M KNO_3	62.3	0.22	3
Ru_xO_y	0.1M Na_2SO_4+ 200mg/L NO_3^--N	73	0.274	4
In-S-G	1M KOH+ 0.1M KNO_3	75	3.74	5
M-NDs/Zr-MOF	0.1M Na_2SO_4+ 500ppm NO_3^-	58.1	4.88	6
Cu/Cu_{2+1}O	0.5M K_2SO_4+ 50mg/L NO_3^--N	87.07	0.576	7
Cu-NPs	0.5M K_2SO_4+ 50ppm NO_3^--N	81.99	0.781	8
Fe/Cu-HNG	1M KOH+ 0.1M KNO_3	92.51	0.018	9
Ar-40/Cu_2O	0.5M Na_2SO_4+ 200ppm NO_3^-	85.78	1.188	10
Cu@CuHHTP	0.5M Na_2SO_4+ 500ppm NO_3^-	67.55	3.68	11
Cu nanosheets	0.1M KOH+ 0.01M KNO_3	99.7	0.39	12
Co_3O_4@NiO	0.5M Na_2SO_4+ 200ppm NO_3^-	54.97	0.118	13

Reference

1. Xu, J.; Zhang, S.; Liu, H.; Liu, S.; Yuan, Y.; Meng, Y.; Wang, M.; Shen, C.; Peng, Q.; Chen, J. Breaking local charge symmetry of iron single atoms for efficient electrocatalytic nitrate reduction to ammonia. *Angew Chem Int Edit* **2023**, *62 (39), e202308044.*

2. Wu, Z.-Y.; Karamad, M.; Yong, X.; Huang, Q.; Cullen, D. A.; Zhu, P.; Xia, C.; Xiao, Q.; Shakouri, M.; Chen, F.-Y. Electrochemical ammonia synthesis via nitrate reduction on Fe single atom catalyst. *Nat Commun* **2021**, *12 (1), 2870.*

3. Wang, Z.; Sun, C.; Bai, X.; Wang, Z.; Yu, X.; Tong, X.; Wang, Z.; Zhang, H.; Pang, H.; Zhou, L. Facile synthesis of carbon nanobelts decorated with Cu and Pd for nitrate electroreduction to ammonia. *ACS Appl. Mater. Interfaces* **2022**, *14 (27), 30969-30978.*

4. Qin, J.; Wu, K.; Chen, L.; Wang, X.; Zhao, Q.; Liu, B.; Ye, Z. Achieving high selectivity for nitrate electrochemical reduction to ammonia over MOF-supported Ru_xO_y clusters. *J Mater Chem* **2022**, *10 (8), 3963-3969.*

5. Lei, F.; Xu, W.; Yu, J.; Li, K.; Xie, J.; Hao, P.; Cui, G.; Tang, B. Electrochemical synthesis of ammonia by nitrate reduction on indium incorporated in sulfur-doped graphene. *Chem Eng J* **2021**, *426, 131317.*

6. Jiang, M.; Su, J.; Song, X.; Zhang, P.; Zhu, M.; Qin, L.; Tie, Z.; Zuo, J.-L.; Jin, Z. Interfacial reduction nucleation of noble metal nanodots on redox-active metal-organic frameworks for high-efficiency electrocatalytic conversion of nitrate to ammonia. *Nano Lett.* **2022**, *22 (6), 2529-2537.*

7. Ren, T.; Ren, K.; Wang, M.; Liu, M.; Wang, Z.; Wang, H.; Li, X.; Wang, L.; Xu, Y. Concave-convex surface oxide layers over copper nanowires boost electrochemical nitrate-to-ammonia conversion. *Chem Eng J* **2021**, *426, 130759.*

8. Xu, Y.; Wang, M.; Ren, K.; Ren, T.; Liu, M.; Wang, Z.; Li, X.; Wang, L.; Wang, H. Atomic defects in pothole-rich two-dimensional copper nanoplates triggering enhanced electrocatalytic selective nitrate-to-ammonia transformation. *J Mater Chem* **2021**, *9 (30), 16411-16417.*

9. Zhang, S.; Wu, J.; Zheng, M.; Jin, X.; Shen, Z.; Li, Z.; Wang, Y.; Wang, Q.; Wang, X.; Wei, H. Fe/Cu diatomic catalysts for electrochemical nitrate reduction to ammonia. *Nat Commun* **2023**, *14 (1), 3634.*

10. Gong, Z.; Zhong, W.; He, Z.; Liu, Q.; Chen, H.; Zhou, D.; Zhang, N.; Kang, X.; Chen, Y. Regulating surface oxygen species on copper (I) oxides via plasma treatment for effective reduction of nitrate to ammonia. *Appl Catal B-Environ Energy* **2022**, *305, 121021.*

11. Zhu, X.; Huang, H.; Zhang, H.; Zhang, Y.; Shi, P.; Qu, K.; Cheng, S.-B.; Wang, A.-L.; Lu, Q. Filling mesopores of conductive metal-organic frameworks with Cu clusters for selective nitrate reduction to ammonia. *ACS Appl. Mater. Interfaces* **2022**, *14 (28), 32176-32182.*

12. Fu, X.; Zhao, X.; Hu, X.; He, K.; Yu, Y.; Li, T.; Tu, Q.; Qian, X.; Yue, Q.; Wasielewski, M. R. Alternative route for electrochemical ammonia synthesis by reduction of nitrate on copper nanosheets. *Appl Mater Today* **2020**, *19, 100620.*

13. Wang, Y.; Liu, C.; Zhang, B.; Yu, Y. Self-template synthesis of hierarchically structured Co_3O_4@ NiO bifunctional electrodes for selective nitrate reduction and tetrahydroisoquinolines semi-dehydrogenation. *Sci. China Mater* **2020**, *63 (12), 2530-2538.*
